# Xenoline-polarized macrophages as a physiologically relevant in vitro model of tumor-associated macrophages in glioblastoma

**DOI:** 10.21203/rs.3.rs-6567445/v1

**Published:** 2025-05-09

**Authors:** Hasan Alrefai, Benjamin Lin, Amr Elkohly, Manoj Kumar, Taylor L. Schanel, Kevin J. Lee, Patricia H. Hicks, Joshua C. Anderson, Gao Guo, Eun-Young Erin Ahn, C. Ryan Miller, Christopher D. Willey

**Affiliations:** The University of Alabama at Birmingham; The University of Alabama at Birmingham; The University of Alabama at Birmingham; The University of Alabama at Birmingham; The University of Alabama at Birmingham; The University of Alabama at Birmingham; The University of Alabama at Birmingham; The University of Alabama at Birmingham; The University of Alabama at Birmingham; The University of Alabama at Birmingham; The University of Alabama at Birmingham; The University of Alabama at Birmingham

## Abstract

Tumor-associated macrophages (TAMs) are the most abundant non-cancerous cell type in glioblastoma (GBM) and heavily influence GBM biology, contributing to tumor progression, therapeutic resistance, immune evasion, and neovascularization. Current *in vitro* models that utilize IL-4/IL-13 stimulation fail to capture the transcriptional and functional heterogeneity of TAMs observed in vivo. In this study, we utilize a serum-free indirect co-culture model with patient-derived xenolines to polarize primary human macrophages and characterize their molecular and functional phenotypes. We demonstrate that xenoline-polarized macrophages diverge from classical M1/M2 states and instead adopt transcriptional signatures reflective of TAM subsets identified from patients. Notably, macrophages polarized with the radiation-therapy selected xenoline, JX14P-RT, exhibited gene expression patterns enriched for interferon response and hypoxia, mirroring recurrent GBM samples. In contrast, JX14P TAMs showed enrichment in phagocytic gene sets. Functional validation of these phenotypes revealed discrepancies between the transcriptionally predicted and observed phenotypes, emphasizing the importance of integrating phenotypic validation in sequencing studies. Altogether, our findings establish xenoline-polarized macrophages as a more physiologically relevant alternative to traditional models, offering a useful model for studying tumor-immune interaction *in vitro*.

## Introduction

GBM is an aggressive and uniformly lethal primary brain tumor with a median survival of 15–18 months, characterized by resistance to traditional therapies [1, 37]. Despite multidisciplinary approaches to treating GBM, including the use of maximally-safe surgical resection, ionizing radiation, temozolomide, Tumor Treating Fields, and various immunomodulatory agents, recurrence is almost inevitable [6, 14, 51]. This is largely driven by a subpopulation of cells called brain tumor-initiating cells (BTICs), which exhibit marked resistance to conventional therapies and are thought to repopulate the tumor following treatment [5, 8].

BTICs engage in multiple reciprocal signaling mechanisms to recruit peripheral monocytes and differentiate and polarize them into TAMs [10, 34, 54, 55]. The first step of this reciprocal interaction is the recruitment of peripheral monocytes via secretion of chemoattractants such as CCL2 (MCP-1) [7, 11, 22, 39]. Colony-stimulating factor 1 (CSF-1/M-CSF), which is constitutively expressed by BTICs, promotes monocyte differentiation into macrophages [11, 41, 50, 53].

GBM secretes a wide range of factors, including extracellular vesicles (EVs) and cytokines, which polarize these macrophages toward a tumor-supportive phenotype. EVs derived from GBM induce TAM secretion of CCL2, IL-6 and VEGF, which promote monocyte recruitment, tumor invasion, and angiogenesis, respectively [7, 9, 34, 39, 60]. In parallel, GBM-derived immunosuppressive cytokines such as IL-10, macrophage-inhibitory cytokine 1 (MIC-1), and TGF-β inhibit macrophage phagocytosis and stimulate TAMs to secrete additional IL-10 and TGF-β, further suppressing T-cell activation and proliferation [42, 57]. TGF-β also promotes the expression of MMP-2, MMP-9, and MMP-14, facilitating ECM remodeling and tumor invasion [28]. Concurrently, GBM secretes proinflammatory cytokines, including IL-1β, IL-6, and IL-8 [47, 59, 65]. These cytokines enhance TAM secretion of IL-6 and CCL2, further promoting invasion, angiogenesis, and monocyte recruitment [17, 39, 47]. Despite this seemingly paradoxical secretion of both pro- and anti-inflammatory cytokines, these pathways act in concert to reshape the tumor microenvironment (TME) to promote immune evasion, vascularization, and invasion [17, 47, 59, 65].

TAMs comprise up to 40% of the total cells in GBM and account for more than 70% of infiltrating immune cells [19, 58]. Given their abundance and substantial influence on GBM biology, TAMs present a compelling target for immunotherapy. However, most clinical trials to date have focused on the adaptive arm of the immune system with limited success, largely due to the redundant expression of immunosuppressive ligands by both GBM and TAMs [12, 35].

**Figure 1:**
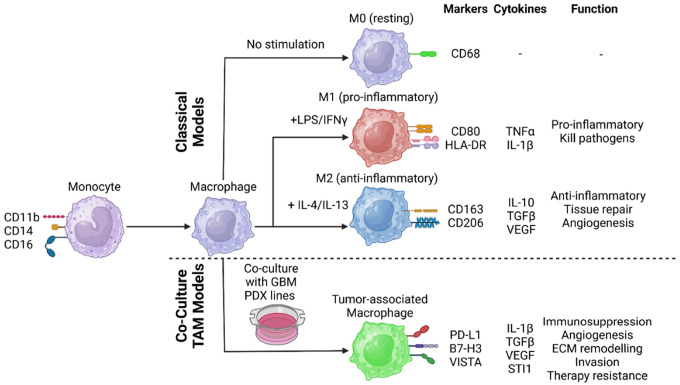
TAM model overview. Schematic summarizing key features of each macrophage polarization state, including stimuli required, markers, cytokine expression, and general function. Created with Biorender.com.

Several single cell studies have revealed that there is a plethora of diverse subsets of macrophages present in the TME with unique gene expression profiles, functional states, and cell surface markers. Despite advancements in the field, many researchers still utilize the archaic M1/M2 dichotomy, effectively ignoring the heterogeneity and plasticity of TAM subsets (Fig. 1). This is largely due to a dearth of high-fidelity *in vitro* models of TAMs. Traditionally, macrophage polarization states have been classified as resting (M0), pro-inflammatory (M1), or anti-inflammatory (M2a-d). The reductionist model states that M1 macrophages may mount an immune response against tumors, and that M2 macrophages promote tumor growth and immunosuppression [33, 48]. Most *in vitro* studies utilize interleukins 4 and 13 to polarize macrophages towards an M2a state to mimic TAMs [23]. While this method is highly reproducible, it does not recapitulate the function or heterogeneity of TAMs seen in vivo [2, 25, 40].

Gupta et al. identified, through single-cell RNA sequencing of primary and recurrent GBM, seven distinct subsets of tumor-infiltrating Monocyte-Derived Macrophages (MDM) and Macrophages (MAC): MAC_Perivascular, MAC_IFN, MDM_Inflam, MAC_Anti-Inflam, MAC_Lipid_Metab, MAC_Metab_Hypoxia, and MDM_Phagocytic [25]. These subsets provide a refined lens through which to assess macrophage heterogeneity beyond the traditional M1/M2 framework. The MAC_Perivascular subset is associated with an anti-inflammatory state and resides within the perivascular niche and is elevated for LYVE1 expression. LYVE1^+^ TAMs promote tumor vascularization by promoting the expansion of perivascular mesenchymal cells [36]. The MAC_IFN and MDM_Inflam subsets both exhibit inflammatory signatures. Specifically, MAC_IFN is defined by the upregulation of Interferon signature genes, which is associated with the mesenchymal phenotype and decreased median survival [31]. The MDM_Inflam subset is marked by elevated expression of SELENOP, which encodes selenoprotein P (SEPP1). This subset corresponds to the SEPP1^hi^ subset identified by Pombo Antunes et al., whose proportion was increased in recurrent GBM [40]. The MAC_Lipid_Metab, MAC_Metab_Hypoxia, and MAC_Anti-Inflam subsets are enriched for hypoxia-related genes. MAC_Lipid_Metab also exhibited signatures associated with lipid metabolism. In contrast, MAC_Metab_Hypoxia is elevated for HMOX1, a marker of myeloid cells that produce IL-10, which drives T-cell exhaustion [43]. The Mac_Anti-Inflam subset exhibits gene signatures associated with chronic inflammatory stimulation and is enriched for EREG, a key regulator of PD-L1 expression, implicating it in immunosuppressive signaling [64]. These hypoxic subsets reflect the functional specialization of TAMs in response to nutrient deprivation and hypoxia, two hallmark features of the GBM TME [46]. Lastly, the MDM_Phagocytic is a subset of tumor-infiltrating macrophages that is defined by increased markers of receptor-mediated endocytosis. These subsets illustrate the pro- and anti-inflammatory spectrum present within the TAM compartment.

Given that BTICs reprogram nearby macrophages through secreted cytokines and extracellular vesicles, we hypothesized that indirect co-culture of macrophages with patient-derived xenograft (PDX) cells grown in BTIC conditions (xenolines) would influence macrophage polarization and function. **In this study we compared the phenotype and function of xenoline-polarized macrophages to classical M0, M1, and M2a states, to discourage continued reliance on IL-4/IL-13-induced M2a macrophages as TAM models.**

Multiple studies have demonstrated that the proportion of some macrophage subtypes differs between primary and recurrent gliomas [25, 40]. These differences are likely driven by the genetic and epigenetic adaptations that occur in response to chemoradiation, resulting in distinct cytokine and metabolite secretion profiles. To mimic the changes seen between primary and recurrent GBM, the Willey and Gillespie labs developed radiation therapy (RT) selected patient-derived xenograft (PDX) models [49]. These models enable investigation of RT-driven adaptations using matched xenolines, thereby minimizing baseline intertumoral variability [38]. **In this study, we assessed how these adaptations influence TAM polarization using our serum-free co-culture system and the paired GBM xenolines.**

## Materials and methods

### Cell culture

#### Patient-derived xenograft (PDX) media

Dulbecco’s Modified Eagle Medium (DMEM)/F12 50/50 (#10–090-CV, Corning, USA), B27 supplement minus Vitamin A (50X) (#12587010, Gibco, USA), 1% penicillin-streptomycin (#30–001-CI, Corning, USA), 1% sodium pyruvate (#5000CI, Corning, USA), EGF (20 ng/mL) (#PHG0311, Gibco, USA), FGF (20 ng/mL) (#PHG0261, Gibco, USA).

#### TheraPEAK^™^ X-VIVO^™^−15 Serum-free Hematopoietic Cell Medium (X-Vivo)

TheraPEAK^™^ X-VIVO^™^−15 Serum-free Hematopoietic Cell Medium (#BEBP02–055Q, Lonza, Switzerland) supplemented with 1% GlutaMAX (#35050–061, Gibco, USA), 1% penicillin-streptomycin (#30–001-CI, Corning, USA), and 1% sodium pyruvate (#5000CI, Corning, USA).

#### GBM patient-derived xenolines.

The JX14P xenoline was originally obtained from Mayo Clinic and maintained in the UAB Brain Tumor Model Core and was approved for use on a UAB IRB approved protocol (IRB-300002910). The matched acquired radiation-resistant PDX line (JX14P-RT) development has been previously described [38, 49]. PDX authentication is routinely confirmed using short tandem repeat (STR). Cell lines are maintained in PDX media as defined above.

### Heat-inactivation of human serum

Matched fresh whole blood was collected in serum clot activator tubes and allowed to clot for an hour. Afterwards, the tubes were centrifuged at 650 × g for 10 minutes, and the serum was collected. The serum was then incubated at 56°C for 30 minutes to heat-inactivate complement factors.

### PBMC isolation and macrophage differentiation

Up to 15 mL of Fresh whole blood from healthy donors was collected in EDTA tubes and diluted with 15 mL phosphate-buffered saline (PBS) in a 50 mL conical tube. 10 mL of Lymphoprep (#18060, StemCell Technologies, Canada) was carefully layered underneath using a 10 mL serological pipette. The tubes were centrifuged at 650 × g for 30 minutes with no brake. The buffy coat was collected and transferred to a new 50 mL tube containing RPMI media (#A1049101, Gibco, USA), then centrifuged for 5 minutes at 650 × g to pellet the cells. The pellet was washed twice with PBS and plated in a 15 cm^2^ dish with RPMI 1640 supplemented with 10% matched donor serum and 50 ng/mL human M-CSF (#11792-HNAH1, SinoBiological, China). The media was replenished with 50 ng/mL of M-CSF on days 3 and 5. On day 7, the cells were washed three times with PBS and lifted for downstream experiments. After replating, the cells were cultured in a 1:1 mixture of PDX and X-VIVO media supplemented with 25 ng/mL M-CSF.

### Macrophage polarization

#### M1 polarization

Resting macrophages were stimulated with 20 ng/mL IFNγ (#300–02-500UG, Gibco, USA) and 1 μg/mL of lipopolysaccharide (#00–4976-03, ThermoFisher, USA) for 24 hours to induce a pro-inflammatory phenotype.

#### M2 polarization

Resting macrophages were stimulated with 20 ng/mL of IL-4 (204-IL, R&D Systems, USA) and IL-13 (#213-ILB, R&D Systems, USA) for 72 hours to induce an anti-inflammatory phenotype.

#### TAM polarization

Resting macrophages were indirectly co-cultured with GBM PDX xenolines using transwell inserts with 3 μm pores (#353096, Corning, USA) for 72 hours, as previously described [4]. Macrophages were seeded in the lower well and GBM was seeded in the upper insert.

### Immunofluorescence staining

1×10^5^ macrophages were polarized toward various states. Afterwards, they were washed three times with PBS. Cells were fixed with 4% PFA for 15 minutes at room temperature. Following fixation, the cells were washed three times with PBS, blocked for one hour in a 5% BSA solution in PBS, and then stained with the fluorescently labeled antibodies αCD80-BV421 (#562844, BD Biosciences, USA), αCD369-BV711 (#745524, BD Biosciences, USA) and αCD206-APC (#550889, BD Biosciences, USA) at a concentration of 1:1000 at 4°C overnight. Afterwards, they were washed three times with PBS and incubated in a PBS solution containing a 1:1000 dilution of CFSE (#C34554, ThermoFisher, USA) for 10 minutes. Coverslips were then mounted onto XCyto10 2-sample slides (#942 − 0010, ChemoMetec, Denmark) using ProLong^™^ Glass Antifade Mountant (#P36980, ThermoFisher, USA). Images were obtained on the Xcyto10 Image Cytometer (ChemoMetec, Denmark). Statistical analysis was performed using a one-way ANOVA (n = 4 per group).

### Conditioned media generation

5×10^5^ macrophages were polarized toward various states. Afterwards, they were washed twice with PBS. 2.2 mL of fresh 1:1 PDX:X-VIVO media supplemented with 25 ng/mL M-CSF was added to each well and the cells were incubated for 24 hours. Afterwards, the media was collected, centrifuged at 1000 × g for 5 minutes, and filtered through a 0.2 μm filter (#09–719C, Fisher Scientific, USA). The resulting conditioned media was divided equally into two portions. The portion used for the cytokine array was spiked with Halt Protease inhibitor (#1861279, ThermoFisher, USA) Phosphatase inhibitor (#P5726–5ML, Sigma-Aldrich, USA), and Halt Phosphatase inhibitor (#1861277, ThermoFisher, USA) at the manufacturers’ suggested concentrations. The other portion was left untreated.

### RNA sequencing

5 × 10^5^ PBMC-derived Macrophages were plated in a 6-well plate. Afterwards, they were either maintained as resting, M0, or polarized towards M1, M2, JX14P-TAM, or JX14P-RT TAM states. Total RNA was extracted using a Qiagen RNeasy mini kit (#74104, Qiagen, Netherlands). Samples were submitted to Novogene (Novogene Co., Ltd., Beijing, China) for DNase digestion, quality control assessment, library preparation, and sequencing. RNA Integrity was assessed using RNA ScreenTape (Agilent Technologies TapeStation), with all RNA integrity numbers greater than 9.0. Ribosomal RNA was depleted before conducting stranded library preparation. Sequencing was performed using biological quadruplicate for each polarization state on a NovaSeq X Plus Series using 150bp paired end reads at 150M read depth. Reads were aligned to the human genome Gencode vH43 using STAR (2.7.10b). BAM files were indexed with Samtools (v1.17). Aligned transcripts were quantified using Salmon (v1.10). Downstream analysis was performed in R after filtering out non-protein coding genes and genes with low reads.

### RNA downstream analysis

Differential expression analysis was performed using DESeq2 (v1.42.1) in R (v4.3.2) using raw counts with batch effects (donor ID) incorporated into the design formula. Differentially expressed genes (DEGs) were identified as having an absolute log2 foldchange (L2FC) > 1 and a false discovery rate (FDR) < 0.05. For visualization, Variance stabilizing transformation (VST) and batch correction using limma (v3.58.1) removeBatchEffects() was applied to raw counts. Principal Component Analysis (PCA) was performed using PCAtools (v2.14.0). Heatmaps displaying the top variable genes were generated from row-wise z-score normalized expression values using ComplexHeatmap (v2.18.0). All unsupervised hierarchical clustering was performed using batch-corrected variance-stabilized transformed expression values. For Gene Set Enrichment Analysis (GSEA), rank lists were generated using the Wald test statistic from DESeq2. Gene set enrichment was assessed using fGSEA (v1.28.0) against the Hallmark (MSigDB) and GlioTIME-36 gene sets. We subset their GlioTime36 matrix to only include gene lists belonging to macrophages. Enrichment results were visualized with ggplot2 (v3.5.1) to generate waterfall and pairwise comparison plots.

### PamStation12 kinomics analysis

Kinome profiling was performed by the UAB Kinome Core as previously described [16, 29]. 5×10^5^ Macrophages were lysed in mammalian protein extraction reagent (MPER) (#78501, ThermoFisher, USA), with Halt Protease Cocktails (#1861279, ThermoFisher, USA) and Phosphatase Inhibitor (#1861277, ThermoFisher, USA) for 15 minutes at 4°C to extract protein for kinomic analysis. A Bicinchoninic acid (BCA) assay was performed to determine protein concentration. 15 μg of protein was loaded per array onto tyrosine kinome PamChips (#86412, Pamgene, The Netherlands). Image processing was performed with Evolve, and signal processing and visualization were performed with BioNavigator (v6.3, PamGene). Network modeling of kinases and other data was conducted with MetaCore (Clarivate Analytics).

### Differential cytokine expression analysis

A RayBiotech GS1 Cytokine Array Kit (#GSH-CYT-1–4, RayBiotech, USA) was used to quantify the relative abundance of 20 distinct cytokines in the conditioned media of macrophages from 4 biological donors polarized toward 5 different states in technical triplicate. The assay was performed according to the manufacturer’s protocol. Slides were imaged using a GenePix 4100A Microarray Scanner (Molecular Devices, USA) with a 532 nm excitation laser and a PMT gain of 500. GenePix Pro software (v7.4.0) was used to extract and quantify the background-subtracted fluorescence intensities (F532 Mean – B532) for each cytokine. The resulting data was tabulated and exported to R (v4.3.2) for analysis. Raw cytokine array data were processed by normalizing signal intensities to the POS2-control on each slide to account for slide-specific effects. The LIMMA (v3.58.1) package was used to identify differentially expressed (DE) cytokines. A design matrix was constructed using the model.matrix function, followed by fitting a linear model with lmFit(). Pairwise contrasts were defined with makeContrasts() and applied to the model using constrasts.fit() and eBayes() to moderate variance. DE cytokines for all contrasts were extracted using topTable(), with p values adjusted by FDR method (q). Significantly differentially expressed cytokines, q < 0.05, are displayed on volcano plots using the EnhancedVolcano package (v1.20.0). Heatmaps were generated using ComplexHeatmap (v2.18.0) on log2(n + 1) transformed normalized intensities. PCA was performed using PCAtools (v2.14.0).

### Tumor microarray gene expression analysis

Patient-matched primary and recurrent IDH-wild type GBM specimens were identified through the UAB Brain Tumor Biorepository, and formalin-fixed paraffin-embedded (FFPE) tissue cores were selected for generation of a tumor microarray (TMA). Spatial transcriptomic profiling was conducted using NanoString’s GeoMx Digital Spatial Profiler (NanoString Technologies, USA), with whole transcriptome coverage. Transcriptomic data processing and quality control were performed using the GeoMxWorkflows (v1.8.0), GeoMxTools (v3.6.2), and NanoStringNCTools (v1.10.1) packages in R (v4.3.2). Low-quality segments and lowly expressed genes were filtered according to manufacturer-recommended guidelines. Normalization, dimensionality reduction, and downstream analyses were conducted using the Bioconductor framework. Differential expression analysis was performed between primary and recurrent GBM samples using mixedModelDE() from the GeoMxWorkflows package. The model formula specified recurrence status as a fixed effect and patient ID as a random intercept: ~ testStatus + (1 | PtID). Least-squares means estimates were extracted for each gene from the lsmeans output and used to compute log fold-change and p-values. Multiple testing correction was performed using the Benjamini–Hochberg method, and genes with a false discovery rate (FDR) < 0.05 were considered significantly differentially expressed. Gene Set Enrichment Analysis (GSEA) was performed on ranked gene as previously described.

### Phagocytosis assay

5×10^4^ macrophages were plated and polarized to each condition in technical quadruplicate in a 24-well plate. GBM cells were stained with 1 ug/mL of pHRhodoRed (#P36600, ThermoFisher, USA). 1×10^5^ GBM PDX cells were plated per well in 400 μL of a 1:1 mixture of PDX and X-VIVO supplemented with 25 ng/mL of M-CSF. Cells were allowed to remain in direct contact for 6 hours. After 6 hours, the media was aspirated, and the wells were gently washed 3 times with PBS. Afterwards, they were fixed in 4% PFA for 15 minutes. Cells were blocked with 5% BSA for 1 hour at room temperature and then stained with an EGFP-conjugated anti-CD45 (#11–9459-42, ThermoFisher, USA) antibody at a concentration of 1:1000 overnight at 4°C. Images were obtained using an Agilent BioTek Cytation 5 Image cytometer (Agilent, USA). % phagocytosis was calculated by dividing the number of double positive cells by CD45^+^ cells. % phagocytosis was normalized to their respective M0 donor controls to account for baseline variability in phagocytosis rates between different donors. Experiments were performed in technical quadruplicate using three separate biological donors. Statistical analysis was performed using a one-way ANOVA (n = 3 per group).

### NLS-EGFP expressing PDX line production

1×10^6^ HEK293T cells were plated on a geltrex-coated 10cm^2^ dish. Cells were transfected with 2 μg of PsPAX2 (Plasmid #12260, Addgene, USA), 4 μg of PMD2.G (Plasmid #12259, Addgene, USA, and 6 μg of the NLS-EGFP vector (Vectorbuilder, USA) for 24 hours using the Lipofectamine 3000 reagent (#L3000015, Invitrogen, USA) in Opti-MEM (#11058, Invitrogen, USA). The lentiviral vector used to overexpress NLS-mEGFP in our study, pLV[Exp]-Puro-CMV + intron > NLS_EGFP, was constructed and packaged by VectorBuilder. The vector ID is VB220505–1225efw, which can be used to retrieve detailed information about the vector on vectorbuilder.com. After 16 hours, media was replaced with 4mL of fresh PDX media. Media was collected and replaced every 24 hours. The media was spun down at 2000g for 10m and the supernatant was collected and pooled. After 72 hours of collection, the media was concentrated using Takara Biosciences Lenti-X Concentrator following the manufacturer’s protocol. The viral pellet was resuspended in 1 mL of PDX media. 100 μL of virus was added to an Eppendorf tube containing 1×10^5^ JX14 or JX14P-RT in a total volume of 500 μL with 8 μg/mL polybrene. A spinfection was performed by spinning the virus-cell mixture at room temperature for 45 minutes at 200g. Afterwards, the supernatant was aspirated, and the cells were resuspended in 5mL of fresh PDX media and plated in a geltrex-coated T25 flask. The cells were expanded and subsequently flow-sorted for EGFP-positive cells.

### Spheroid invasion assay

JX14P-RT NLS-EGFP cells were dissociated and allowed to rest for 48 hours. Once they formed small spheroids, 50% CM or fresh media was mixed with fresh PDX:X-Vivo media supplemented with 25 ng/mL M-CSF and then combined with Matrigel (#354230, Corning, USA) at a 2:1 (v/v) ratio to embed the spheroids. Regions of Interest (ROIs) were defined using an Agilent BioTek Cytation5 fluorescence widefield microscope. Images were acquired every 6 hours over a 72-hour period and exported as .tif for analysis in ImageJ. Spheroid area was quantified using a custom ImageJ macro applied to time-lapse .tif image sets. For each set, the user manually selected a rectangular region of interest (ROI) encompassing the spheroid in the first timepoint (t = 0). This ROI was applied to all subsequent frames in the series. Prior to measurement, rolling ball background subtraction was performed on each frame. Images were then thresholded using Otsu’s method, and a binary mask was generated within the ROI. The spheroid area was measured at each timepoint. To account for variability in the starting size of each spheroid, area was normalized to t = 0 for each spheroid. To assess the effects of polarization state and time on normalized spheroid area, a linear mixed-effects model was fitted using the lmer function from the lme4 package (v1.1–36) in R (4.3.2). The model included fixed effects for polarization, time, and their interaction, with time included as a random intercept term to account for repeated measures: [Normalized_Area ~ Time * Polarization + (1 | Time)]. Post-hoc pairwise comparisons between polarization groups were performed at each individual timepoint using the emmeans package (v1.10.7), with Tukey post-hoc correction for multiple comparisons (n = 9–37 per group). Results are displayed as Mean ± SEM.

### Angiogenesis assay

60μL of ice cold Matrigel was placed inside a 96 well plate using prechilled pipette tips and allowed to solidify for 30min at 37*C. Afterwards, 2.5×10^4^ HUVEC cells were plated inside each well in 160μL of un-supplemented Human Large Vessel Endothelial Cell Basal Medium (#M200PRF500, Gibco, USA). 40μL of conditioned media was added to each well. Images were taken at 6 hours using an Agilent BioTek Cytation 5 fluorescence widefield microscope. The number of honeycombs were counted in a blinded manner for each image. Statistical analysis was performed using a one-way ANOVA (n = 3 per group).

### Quantification and statistical analysis

The specific quantification methods of corresponding items are described above. Statistical analysis of IF, phagocytosis, angiogenesis data were performed using GraphPad Prism v10.4.0 (USA). Multiple group comparisons were conducted using one-way, for IF and angiogenesis, or two-way, for phagocytosis, ANOVA followed by post hoc Tukey’s multiple comparisons test. These data are presented as Mean ± SD. All other analysis were performed in R (v4.3.2) as described above. Invasion data are presented as Mean ± SEM. * *P* < 0.05, ** *P* < 0.01, *** *P* < 0.001, **** *P* < 0.0001.

## Results

THP-1-derived M2 macrophages fail to fully mimic canonical M2 phenotypes. We previously established a serum-free co-culture system with THP-1 macrophages [3, 4, 27]. We utilized this system to compare the molecular phenotype of co-culture polarized THP-1 macrophages with classically polarized M1 and M2 THP-1 macrophages. We previously demonstrated that LPS/IFNγ-polarized THP-1 macrophages upregulate the traditional M1 markers, CD80 and HLA-DR [27]; however, IL-4/IL-13 polarized THP-1 macrophages did not upregulate CD206 (P = 0.6167) **(Fig. S1A-B)**, a canonical M2 marker frequently used to identify TAMs *in vitro* [20, 23, 36, 52, 63]. We confirmed that LPS/IFNγ-stimulated THP-1 macrophages upregulated the M1-associated cytokines IL-1β and TNFα on both RNA (TNFα *P* < 0.0001; IL-1 β, P < 0.0001) and protein (TNFα P < 0.0001; IL-1β, *P* < 0.0001) levels relative to M0 **(Fig. S1C-D, F-G)**. There was a mild increase in IL-10 on the transcriptional level (*P* < 0.0001), and a mild decrease on the protein level (*P* < 0.0001) **(Fig. S1E-G)**. M2 THP-1 macrophages upregulated IL-4 and VEGF on the protein level (IL-4 *P* < 0.0001; VEGF *P* < 0.0001), confirming successful IL-4/IL-13 stimulation. Then, we polarized THP-1 macrophages by indirect co-culture with the radiation-resistant xenoline, JX39P-RT. Interestingly, the cytokine expression profile of JX39P-RT polarized TAMs reflected a hybrid phenotype, exhibiting both M1-like (MMP9) and M2-like (VEGF) features, suggesting deviation from the M1/M2 dichotomy **(Fig. S1C-D, F-G)**. We performed phenotypic characterization on the different polarization states of these macrophages. M2-polarized macrophages had the greatest phagocytic capacity against the xenolines JX39P and its matched radiation-therapy selected counterpart, JX39P-RT (39P, M2 vs all other groups *P* < 0.01; 39P-RT M2 vs all other groups *P* < 0.005). No other polarization state showed significant differences in phagocytosis **(Fig. S1H-I)**. Conditioned media (CM) from M1-polarized macrophages had the greatest invasion-promoting capacity up to 72 hours in a transwell invasion assay (*P* < 0.05). There was no significant difference between M0, M2, JX39P-TAM or JX39P-RT TAM CM (P > 0.9995) **(Fig. S1J)**. Based on the limited proteomic and transcriptomic profiling, the xenoline-polarized TAMs did not distinctly resemble either the M1 or M2 polarization state. In fact, they most closely resemble the M0 state, corroborating previous reports [21]. Moreover, consistent with previous reports, THP-1 macrophages failed to fully express markers associated with M2 polarization, despite increasing M2-associated cytokine transcription, thereby limiting their utility as a comparative model [45]. Given these limitations, we turned to primary peripheral blood monocyte (PBMC)–derived macrophages to more accurately model distinct polarization states for comparative analysis.

**Figure 2:**
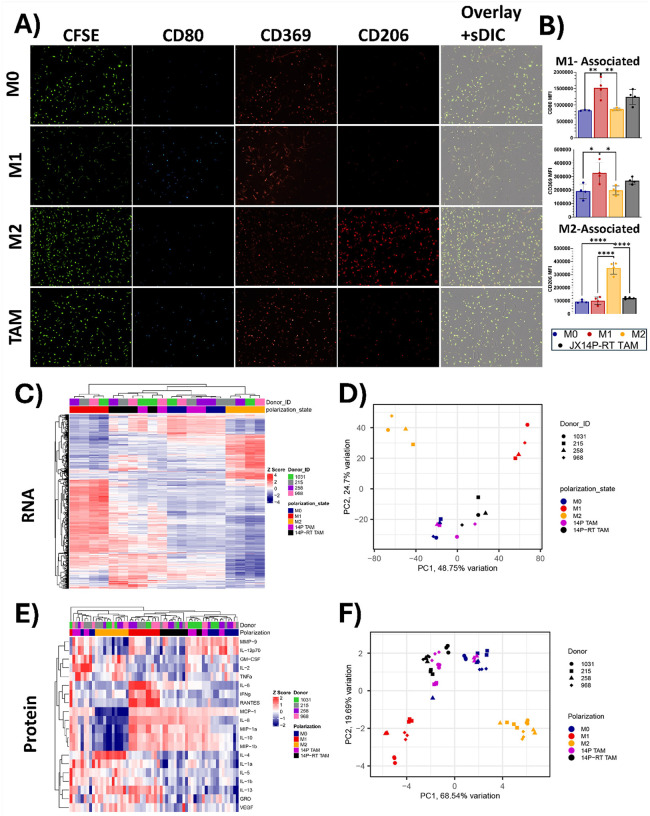
The M1/M2 dichotomy is insufficient for characterizing macrophages polarized by xenolines. **(A)** Representative images of macrophages probed for CD80, CD369, or CD206 following stimulation with LPS/IFNγ, IL-4/IL-13, or co-culture with JX14P-RT. **(B)** Quantification of mean fluorescent intensity (MFI) of CD80, CD369, and CD206. **(C)** Unsupervised heatmap of the top 500 variable genes for macrophages polarized to M0, M1, M2, 14P TAM, and 14P-RT TAM states. **(D)** Principal component analysis of macrophages polarized to M0, M1, M2, 14P TAM, and 14P-RT TAM states. **(E)** Unsupervised heatmap of 20 cytokines secreted by macrophages polarized to M0, M1, M2, 14P TAM, and 14P-RT TAM states. **(F)** Principal component analysis of macrophages polarized to M0, M1, M2, 14P TAM, and 14P-RT TAM states. * *P*<0.05, ** *P*<0.01, **** *P*<0.0001

The M1/M2 dichotomy is insufficient for characterizing macrophages polarized by xenolines. Because the M2-polarization of THP-1 macrophages did not upregulate CD206, we polarized human PBMC-derived macrophages into M0, M1, and M2 using classical stimuli, as well as TAMs by indirectly co-culturing them with the radiation-resistant xenoline JX14P-RT which retain their radiation-resistant phenotype *in vitro* **(Fig. S2)**. As expected, PBMC-derived macrophages stimulated with LPS/IFNγ upregulated the M1-associated markers CD80 and CD369 relative to naïve/resting macrophages and IL-4/IL-13-stimulated macrophages (CD80 *P* < 0.005, CD369 *P* < 0.05) (Fig. 2A–B). Interestingly, the expression of these M1-associated markers did not significantly differ between M1-polarized macrophages and JX14P-RT–polarized TAMs (CD80 *P* = 0.2668; CD369 *P* = 0.4215). As expected, IL-4/IL-13-stimulated macrophages upregulated the M2-associated marker CD206 relative to all other polarization states (*P* < 0.0001) (Fig. 2A–B). To further characterize the differences among these polarization states, we performed bulk RNA sequencing on PBMC-derived macrophages from four different donors polarized toward classical M0, M1, and M2 states as well as toward the JX14P and JX14P-RT TAM phenotypes. An anonymized donor identifier was included as a covariate in our design formula to account for inter-donor immunological variability. Unsupervised hierarchical clustering of the top 500 variable genes revealed that the polarization state strongly drives the transcriptional heterogeneity of PBMC-derived macrophages (Fig. 2C). Principal component analysis (PCA) demonstrated that the first two principal components accounted for ~ 73% of the total variance (Fig. 2D). PC1 (49% variance) separated xenoline-polarized TAMs and M0 from classical M1 and M2 states. Notably, JX14P-TAMs clustered with the M0 state. In contrast, JX14P-RT TAMs clustered distinctly from M0 with partial overlap with JX14P TAMs. We next characterized the secretome of these macrophage polarization states using the RayBiotech GS1 cytokine array, which semi-quantitatively detects 20 analytes. Unsupervised hierarchical clustering showed that the polarization state significantly alters the cytokine secretion patterns of PBMC-derived macrophages (Fig. 2E). PCA of GS1 cytokine array data revealed that the first two principal components accounted for ~ 88% of total variance, with PC1 (69% variance) distinguishing xenoline-polarized macrophages and M0 from classical M1 and M2 states. M1 and M2 macrophages clustered distinctly, reflecting differences in their cytokine expression profiles. JX14P and JX14P-RT TAMs shared similar cytokine profiles. These results indicate that the variation of the secretome is primarily driven by polarization state, and that TAMs polarized by xenolines do not align with either M1 or M2 macrophages. Together, these findings confirm that PBMC-derived macrophages respond robustly to classical M1 and M2 stimuli. Moreover, xenoline-polarized macrophages exhibit transcriptional and secretory profiles that diverge from canonical polarization states, further challenging the binary M1/M2 framework frequently used in TAM studies.

**Figure 3:**
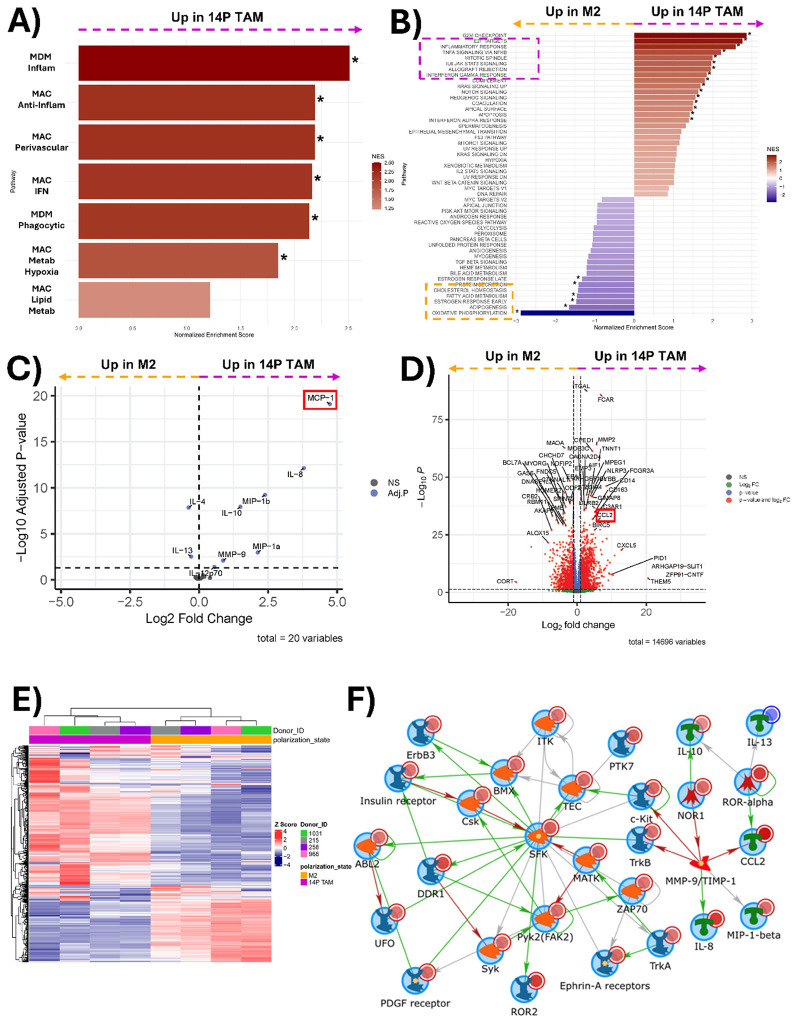
Macrophages polarized by JX14P exhibit distinct transcriptomic, kinomic, and cytokine expression profiles from M2 macrophages. **(A)** Pairwise plot of GSEA enrichment scores of 14P TAMs relative to M2 macrophages for the macrophage-subset of the GlioTime-36 matrix. **(B)** Pairwise plot of GSEA enrichment scores of 14P TAMs relative to M2 macrophages for the hallmark gene sets. * indicates P<0.05 in **A**) and **B**). **(C)** Volcano plot visualization of differentially secreted cytokines between 14P TAMs and M2 macrophages. **(D)** Volcano plot visualization of differentially expressed genes between 14P TAMs and M2 macrophages. **(E)** Unsupervised heatmap of the top 500 variable genes for 14P TAMs and M2 macrophages. **(F)** Network of RNA-seq and kinomic alterations in macrophages polarized to a JX14P TAM state compared to M2 macrophages. Altered RNA-seq genes (LFC>5.0; pAdj<0.005) and altered kinases (mean final score >2.0) were combined and analyzed via GeneGo MetaCore to generate literature annotated networks. Networks used an Auto-expand model with a maximum size (node n) of 75. Light red or light blue circles in the top right of each symbol indicate kinomically identified kinases and cytokines as increased (14P TAM>M2) or decreased, respectively. Dark red or dark blue circles indicate RNA-seq identification. Arrow heads between nodes indicate directionality and general type of interaction (green, positive/activating; red, negative/inhibitory; grey, other).

Macrophages polarized by JX14P exhibit distinct transcriptomic, kinomic, and cytokine expression profiles from M2 macrophages. GSEA using the subsets identified by Gupta *et al*. revealed significant enrichment in JX14P TAMs compared to M2 macrophages in all pathways except for MAC_Lipid_Metab (Fig. 3A). GSEA of hallmark pathways highlight enrichment of pathways associated with inflammatory responses in JX14P TAM (marked in magenta) (Fig. 3B). Interestingly, M2 macrophages were enriched for hallmark pathways related to lipid metabolism and oxidative phosphorylation (marked in yellow) (Fig. 3B). Differential cytokine expression analysis revealed an upregulation of IL-6, IL-8, MIP-1α, MIP-1β, MMP-9, and MCP-1 (CCL2) in JX14P TAMs and a downregulation of IL-4 and IL-13 relative to M2 macrophages (Fig. 3C), suggesting that JX14P TAMs may have a higher capacity for tumor-promoting inflammation, monocyte recruitment, and invasion relative to M2 TAMs. Gene expression analysis revealed numerous significantly differentially expressed genes (DEGs) between JX14P TAM and M2 macrophages. Of interest, CCL2, CXCL5, and MMP2 were upregulated in JX14P TAM relative to

M2 macrophages (Fig. 3D). Conversely, ALOX15 and CORT were more highly expressed in M2 macrophages (Fig. 3D). Unsupervised hierarchical clustering of the top 500 variable genes showed complete segregation of JX14P TAM and M2 macrophages, demonstrating their distinct transcriptomic profiles (Fig. 3E). Kinomic profiling revealed a global elevation in kinase signaling in JX14P TAMs vs M2 macrophages **(Fig. S3A)**. To illustrate functional interactions, we integrated the altered cytokine, RNA, and kinomic datasets to construct biological networks. Network modeling revealed a Src family kinase (SFK)-centric signaling hub that included IL-8, IL-10, CCL2 (Fig. 3F). Comparative analyses were extended to include JX14P TAMs versus M0 and M1 macrophages, revealing additional differences in transcriptomic and cytokine expression profiles **(Fig. S4–5)**.

**Figure 4:**
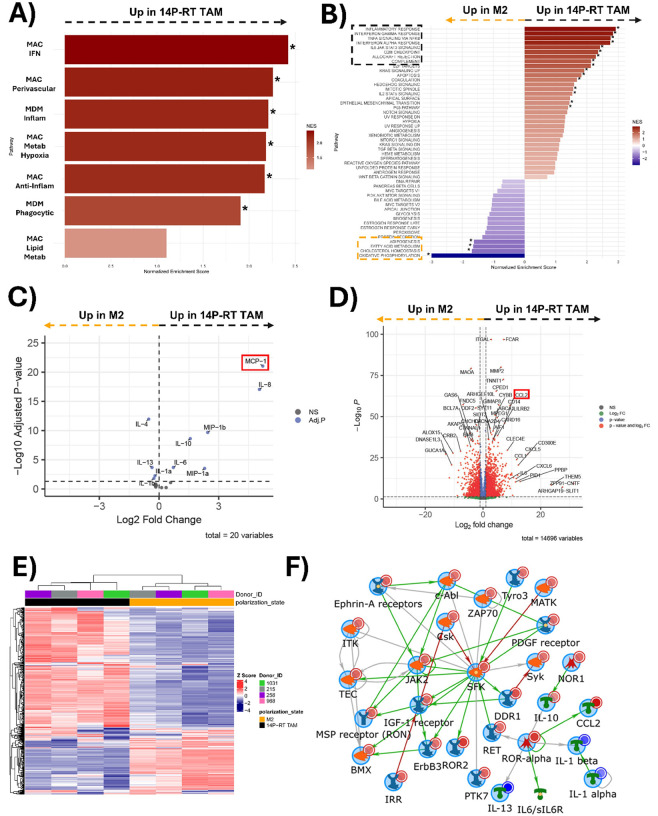
Macrophages polarized by JX14P-RT exhibit distinct transcriptomic, kinomic, and cytokine expression profiles from M2 macrophages. **(A)** Pairwise plot of GSEA enrichment scores of 14P-RT TAMs relative to M2 macrophages for the macrophage-subset of the GlioTime-36 matrix. **(B)** Pairwise plot of GSEA enrichment scores of 14P-RT TAMs relative to M2 macrophages for the hallmark gene sets. * indicates *P*<0.05 in **A**) and **B**).**(C)** Volcano plot visualization of differentially secreted cytokines between 14P-RT TAMs and M2 macrophages. **(D)** Volcano plot visualization of differentially expressed genes between 14P-RT TAMs and M2 macrophages. **(E)**Unsupervised heatmap of the top 500 variable genes for 14P-RT TAM and M2 macrophages. **(F)** Network of RNA-seq and kinomic alterations in macrophages polarized to a JX14P-RT TAM state compared to M2 macrophages. Altered RNA-seq genes (LFC>5.0; pAdj<0.005) and altered kinases (mean final score >2.0) were combined and analyzed via GeneGo MetaCore to generate literature annotated networks. Networks used an Auto-expand model with a maximum size (node n) of 75. Light red or light blue circles in the top right of each symbol indicate kinomically identified kinases and cytokines as increased (14P-RT TAM>M2) or decreased, respectively. Dark red or dark blue circles indicate RNA-seq identification. Arrow heads between nodes indicate directionality and general type of interaction (green, positive/activating; red, negative/inhibitory; grey, other).

Macrophages polarized by JX14P-RT exhibit distinct transcriptomic, kinomic, and cytokine expression profiles from M2 macrophages. GSEA using Gupta *et al.’s* macrophage subset signatures revealed significant enrichment in JX14P-RT TAMs compared to M2 macrophages in all pathways except for MAC_Lipid_Metab (Fig. 4A). GSEA of hallmark pathways highlights enrichment of inflammatory response-related pathways in JX14P-RT TAM (marked in black) (Fig. 4B). While JX14P TAMs and JX14P-RT TAMs shared many enriched pathways relative to M2 macrophages, JX14P-RT TAMs showed additional enrichment in pathways related to interferon alpha signaling and epithelial-to-mesenchymal transition (Fig. 4B). M2 macrophages exhibited an enrichment of hallmark pathways related to lipid metabolism and oxidative phosphorylation relative to JX14P-RT TAMs (marked in yellow) (Fig. 4B). Differential cytokine expression analysis revealed an upregulation of IL-6, IL-8, IL-10, MIP-1α, MIP-1β, and MCP-1 (CCL2) in JX14P-RT TAMs relative to M2 and downregulation of IL-4, IL-13, IL-1β, and IL-1α (Fig. 4C), suggesting that JX14P-RT TAMs have a higher capacity for tumor-promoting inflammation and monocyte recruitment [7, 39]. Gene expression analysis revealed numerous significantly differentially expressed genes between JX14P-RT TAM and M2 macrophages. Interestingly, CCL2, CXCL5, and MMP2 were also upregulated in JX14P-RT TAMs relative to M2 (Fig. 4D). Unsupervised hierarchical clustering of the top 500 variable genes showed complete segregation between JX14P-RT TAMs and M2 macrophages, demonstrating their distinct transcriptomic profiles (Fig. 4E). Kinomic profiling revealed a global elevation in kinase signaling in JX14P-RT TAMs vs M2 macrophages. **(Fig. S3B)** To visualize functional interactions, we combined the altered cytokine, RNA, and kinomic data to generate biological networks. Network modeling revealed a Src Family Kinase (SFK)-centric network that included IL-10 and CCL2 (Fig. 4F). Comparative analyses were extended to include JX14P-RT TAMs versus M0 and M1 macrophages, revealing additional differences in transcriptomic and cytokine expression profiles **(Fig. S6–7).**

**Figure 5:**
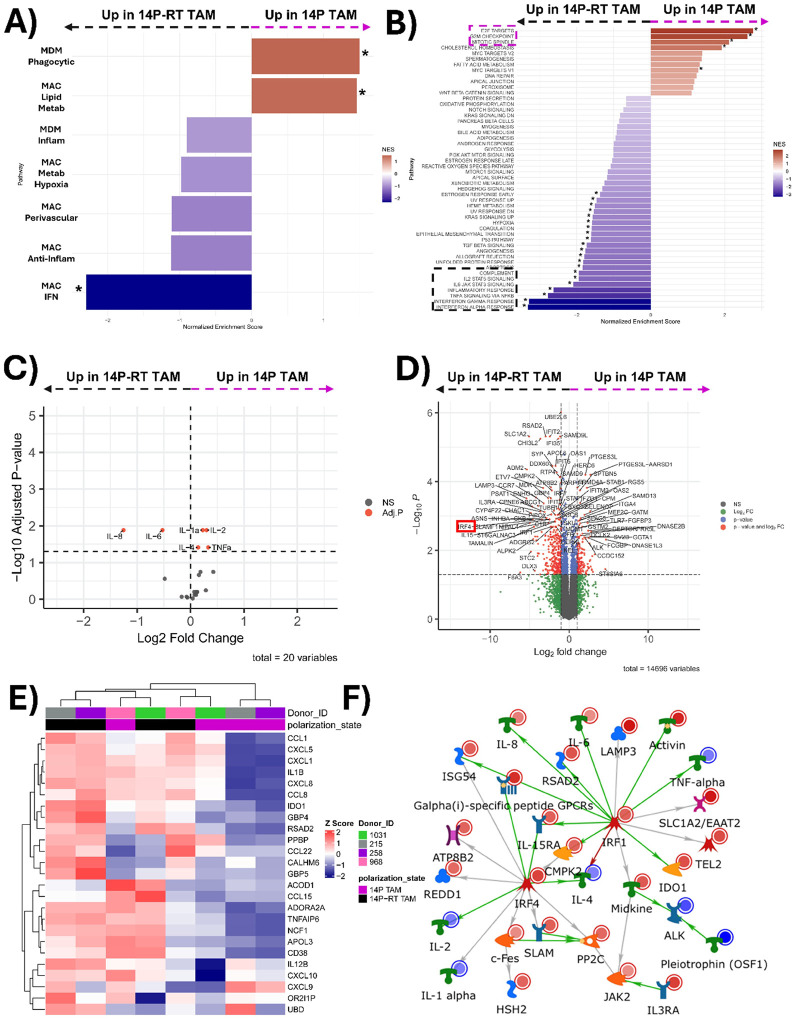
Macrophages polarized by JX14P exhibit distinct transcriptomic, kinomic, and cytokine profiles compared to those polarized by the RT-selected xenoline JX14P-RT. **(A)** Pairwise plot of GSEA enrichment scores of 14P TAMs relative to 14P-RT TAMS for the macrophage-subset of the GlioTime-36 matrix. **(B)** Pairwise plot of GSEA enrichment scores of 14P TAMs relative to 14P-RT TAMs for the hallmark gene sets. * indicates *p* < 0.05 in **A**) and **B**). **(C)** Volcano plot visualization of differentially secreted cytokines between 14P TAMs and 14P-RT TAMs. **(D)** Volcano plot visualization of differentially expressed genes between 14P TAMs and 14P-RT TAMs. **(E)** Unsupervised heatmap of the top 25 variable genes for macrophages polarized to 14P TAMs and 14P-RT TAMs. **(F)** Network of RNA-seq and kinomic alterations in macrophages polarized to a JX14P-RT TAM state compared to 14P TAMs. Altered RNA-seq genes (LFC>2.0; pAdj<0.005) and altered kinases (mean final score >2.0) were combined and analyzed via GeneGo MetaCore to generate literature annotated networks. Networks used an Auto-expand model with a maximum size (node n) of 120. Light red or light blue circles in the top right of each symbol indicate kinomically identified kinases and cytokines as increased (14P-RT TAM>14P TAM) or decreased, respectively. Dark red or dark blue circles indicate RNA-seq identification. Arrow heads between nodes indicate directionality and general type of interaction (green, positive/activating; red, negative/inhibitory; grey, other).

Macrophages polarized by JX14P exhibit distinct transcriptomic, kinomic, and cytokine profiles compared to those polarized by the RT-selected xenoline JX14P-RT. GSEA of the TAM subsets identified by Gupta *et al*. revealed significant enrichment of the MDM_Phagocytic and MAC_Lipid_Metab signatures in JX14P TAMs relative to JX14P-RT TAMs. Conversely, the MAC_IFN signature was elevated in JX14P-RT TAMs compared to JX14P TAMs (Fig. 5A). GSEA of hallmark pathways highlights significant enrichment of the E2F targets, G2M checkpoint, and mitotic spindle pathways in JX14P TAMs relative to JX14P-RT TAMs (marked in magenta) (Fig. 5B). In contrast, JX14P-RT TAMs exhibited enrichment of inflammatory response pathways (marked in black), TGFβ signaling, KRAS signaling, UV damage response, and epithelial to mesenchymal transition (Fig. 5B). Cytokine expression analysis revealed upregulation of IL-1α, IL-2, IL-4, and TNFα and downregulation of IL-6 and IL-8 in JX14P TAMs compared to JX14P-RT TAMs (Fig. 5C), suggesting that JX14P-RT TAMs have a higher capacity for tumor-promoting inflammation. Gene expression analysis revealed numerous significant DEGs between JX14P TAMs and JX14P-RT TAMs. Of interest, CCDC152 and SELENOP, also known as SEPP1, were upregulated in JX14P TAM relative to JX14P-RT (Fig. 5D). Interestingly, this contradicts the findings of Pombo Antunes et al. who identified a SEPP1^hi^ population of macrophages that was elevated in recurrent GBM, although their study did not utilize matched primary and recurrent samples. Conversely, IRF4, CCR7, and IFIT2 were upregulated in JX14P-RT TAMs relative to JX14P TAMs (Fig. 5D). Unsupervised hierarchical clustering of the top 25 variable genes revealed moderate segregation of JX14P TAMs and JX14P-RT TAMs, though some overlap was observed. This similarity is expected, given that both groups were polarized using matched xenolines (Fig. 5E). Kinomic profiling revealed increased activity of kinases including JAK2, ITK, and Fes in JX14P-RT TAMs relative to JX14P TAMs **(Fig. S3C)**. To visualize functional interactions, we combined the altered cytokine, RNA, and kinomic data into biological networks. Network modeling revealed an IRF1 and IRF4-centric network that included IL-6 and IL-8.

**Figure 6:**
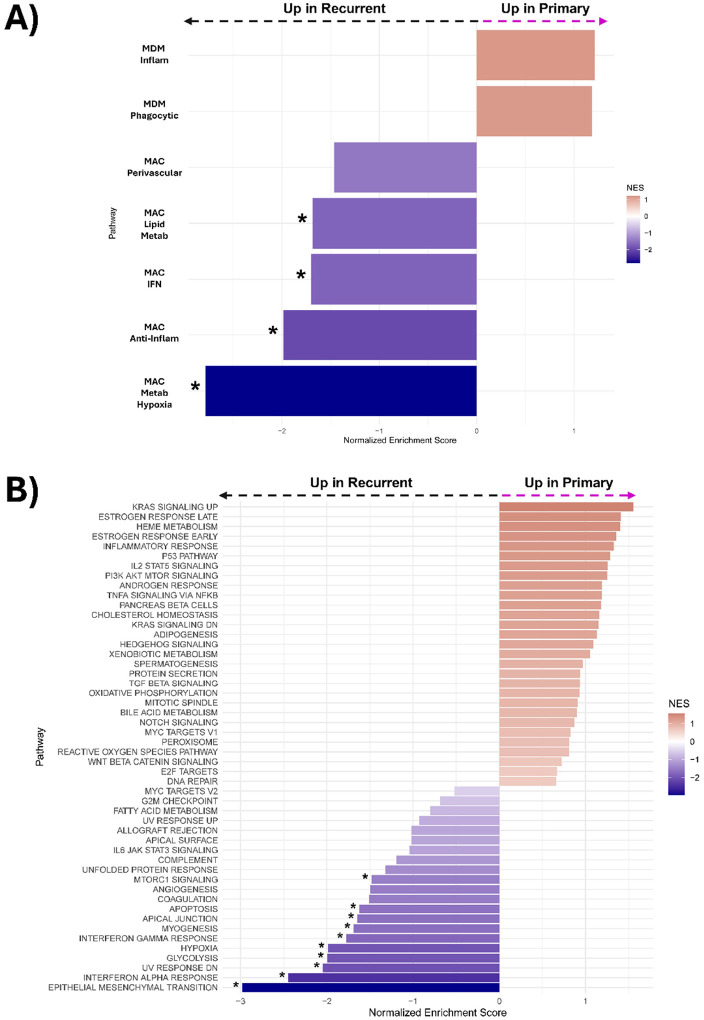
Glioblastoma exhibits increases in many macrophage-specific gene signatures following recurrence. **(A)** Pairwise plot of GSEA enrichment scores of primary relative to recurrent IDH-wild type glioblastoma for the macrophage-subset of the GlioTime-36 matrix. **(B)** Pairwise plot of GSEA enrichment scores of primary relative to recurrent IDH-wild type glioblastoma for the hallmark gene sets. * indicates *P*<0.05 in **A**) and **B**).

Glioblastoma exhibits increases in many macrophage-specific gene signatures following recurrence. Patient-matched primary and recurrent GBM samples were used to generate a tumor microarray (TMA), which was subsequently characterized by using digital spatial transcriptomic profiling. The array represents 10 IDH-wildtype patients at the time of initial resection and again following recurrence. GSEA of the subsets identified by Gupta et al. revealed that these tumors exhibited enrichment of the MAC_Metab_Hypoxia, MAC_Anti-Inflam, MAC_IFN, and MAC_Lipid_Metab subsets at the time of recurrence (Fig. 6A). GSEA of the hallmark gene sets revealed enrichment of the EMT transition, Interferon alpha and gamma response, glycolysis, and hypoxia gene sets at the time of recurrence (Fig. 6B).

**Figure 7:**
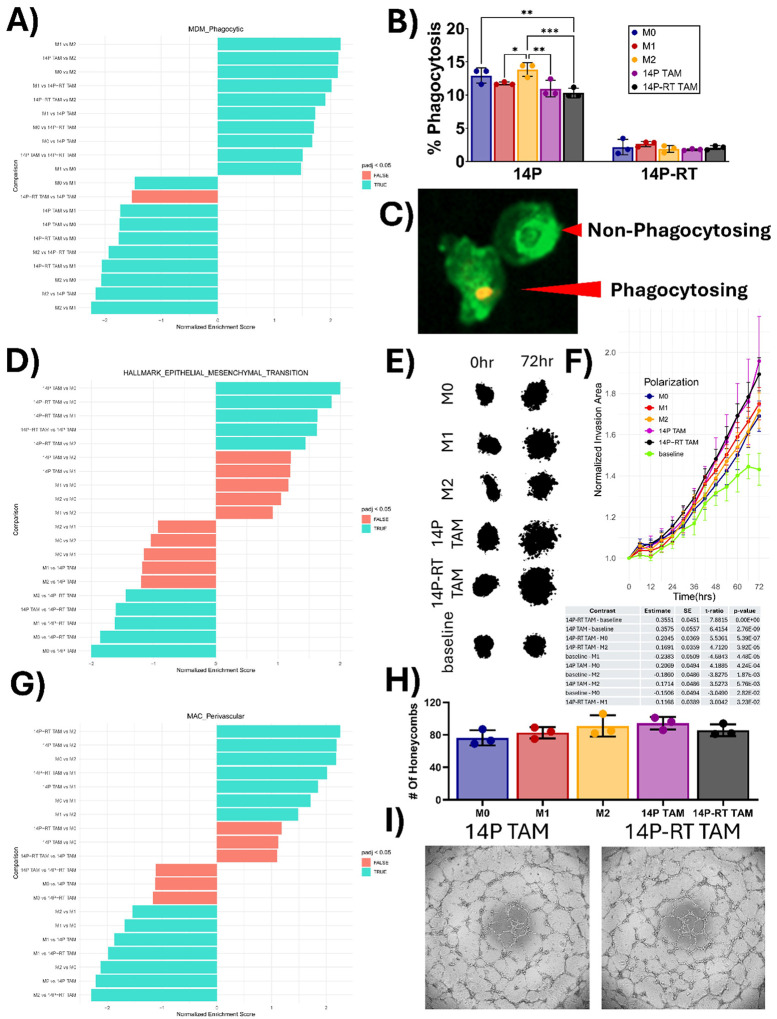
Functional assays reveal subtle differences between the polarization states. **(A)** Waterfall plot showing enrichment scores for the MDM_Phagocytic subset from the macrophage-subset of the GlioTime-36 matrix. **(B)** Quantification of the phagocytosis assay against 14P and 14P-RT for the M0, M1, M2, 14P TAM, and 14P-RT TAM polarization states. Data are shown as mean ± SD. **(C)** Representative images depicting a phagocytosing and non-phagocytosing macrophage. **(D)** Waterfall plot showing enrichment scores for the hallmark EMT gene set. **(E)** Representative images of the spheroid invasion assay at t=0 and t=72 hours. **(F)** Line plot depicting the quantification of the spheroid invasion assay over time. Data are displayed as mean ± SEM. Significant p values at t = 72hrs are shown in the accompanying table. **(G)** Waterfall plot showing enrichment scores for the MAC_Perivascular subset from the macrophage-subset of the GlioTime-36 matrix. **(H)** Quantification of the number of capillary-like structures formed in the HUVEC tube formation assay. Data are shown as mean ± SD. **(I)** Representative images of the tube formation assay following stimulation with 14P TAM and 14P-RT TAM. * *p* < 0.05, ** *p* < 0.01, *** *p* < 0.001

Functional assays reveal subtle differences between the polarization states. To validate these transcriptional signatures, we performed a series of functional assays to assess the behavior of each polarization state *in vitro*. GSEA of the MDM Phagocytic subtype gene set predicted that M1 macrophages would exhibit the greatest phagocytic capacity and that M2 would have the lowest (Fig. 7A). Despite these predictions, M2 macrophages exhibited greater phagocytosis of JX14P cells compared to M1 (P = 0.0327), JX14P TAMs (P = 0.005), and JX14P-RT TAMs (P = 0.0003). Similarly, M0 macrophages showed elevated phagocytic activity against JX14P relative to JX14P-RT TAMs (P = 0.006). There were no significant differences in phagocytosis of JX14P-RT cells across polarization states (P > 0.65). Notably, the baseline phagocytosis rate of JX14P-RT was approximately 3-fold lower than that of JX14P, suggesting that radiation selection may also confer resistance to phagocytosis (Fig. 7B–C). The epithelial to mesenchymal transition (EMT) hallmark gene set was used as a proxy for invasive potential because it is associated with increased ECM remodeling, invasiveness, and motility. GSEA of the EMT hallmark gene set predicted that JX14P-RT TAMs would have the highest invasion promoting capacity (Fig. 7D). To assess this, we performed a spheroid invadopodia assay utilizing the fluorescently labeled xenoline JX14P-RT NLS-EGFP. Conditioned media (CM) from JX14P-RT TAMs significantly promoted more invasion than CM from M0 (*P* < 0.0001), M1 (*P* < 0.05), and M2 (*P* < 0.0001) macrophages; however, no significant differences were observed between CM from JX14P-RT TAMs and JX14P TAMs (*P* > 0.9995) (Fig. 7E–F). Additionally, CM from 14P TAMs significantly promoted more invasion compared to M0 (*P* < 0.0005) and M2 (*P* < 0.01). GSEA of the MAC_Perivascular gene set predicted increased angiogenic potential in JX14P and JX14P-RT.

## DISCUSSION

Advances in GBM research have been hindered by limitations of preclinical models that fail to accurately replicate the tumor’s complex microenvironment. Traditional macrophage models often lack the cellular diversity and structural organization seen in GBM, leading to discrepancies between preclinical findings and clinical outcomes. Of particular concern is the widespread use of IL-4/IL-13 polarized macrophages, a reductionist model that fails to replicate the functional diversity and heterogeneity of TAMs that we see *in vivo* [2, 25, 40]. In this study we demonstrate that macrophages polarized via indirect co-culture with GBM xenolines exhibit unique transcriptional profiles that diverge from the M1 **(Fig. S5 & 7)** and M2 phenotypes (Fig. 3–4). Our findings align with an earlier report suggesting that TAMs, broadly, resemble the M0 phenotype [21]. However, there are several differences between TAMs and the M0 phenotype **(Fig. S4 & 6)**. Both JX14P TAMs and JX14P-RT TAMs displayed enrichment of hallmark signatures related to interferon response, hypoxia, and EMT, while showing decreases in signatures related to lipid metabolism. Additionally, both TAM subsets exhibited increased expression of the pro-inflammatory cytokine IL-8 and anti-inflammatory cytokine IL-10 relative to M0, highlighting the seemingly paradoxical secretion of pro- and anti-inflammatory cytokines that work in concert to promote GBM growth. Interestingly, both JX14P and JX14P-RT TAMs exhibited increased IL-6 secretion relative to IL-4/IL-13-polarized M2a macrophages. Their cytokine profiles align more closely with the M2d phenotype, a TAM-like state induced by IL-6 and Leukemia Inhibitory Factor, rather than the classically described M2a phenotype [15, 56], further bringing the utility of M2a macrophages as TAM models into question. Moreover, while both xenoline-polarized macrophage populations exhibited divergence from M2a macrophages, they also differed significantly from one another. These findings argue against a “one-size fits all” approach and instead support a tailored, xenoline-specific modeling strategy that reflects tumor-intrinsic influences on macrophage polarization.

Recent studies have identified many promising innate immunomodulatory therapeutic targets using these reductionist models. For example, CD47 blockade and disruption of the APP-CD74 axis have been shown to enhance the phagocytosis of GBM cells *in vitro* [24, 32]. However, it is important to note that these results were obtained using resting macrophages, which not only have elevated phagocytic signatures relative to both of our TAM models but also exhibit significantly enhanced phagocytosis relative to JX14P-RT TAMs against the xenoline JX14P (Fig. 6A–B). Using macrophage models that are inherently primed for phagocytosis may lead to the identification of therapeutic targets that ultimately fail in clinical trials due to exaggerated effects observed *in vitro*. To address these modeling limitations, it is imperative to adopt more physiologically relevant models that better capture TAM heterogeneity in GBM.

While murine models such as GL261 have been instrumental in advancing our understanding of GBM biology and immune regulation, they fail to recapitulate key aspects of GBM biology. Syngeneic murine models like GL261 and CT2A were developed through chemical mutagenesis and harbor mutations that are infrequently observed in human GBM [18, 44]. GL261, in particular, is immunologically “hot” due to its high mutational burden and elevated MHC class I expression, making it poorly representative of the immunosuppressive TME characteristic of GBM [30]. Notably, many early studies targeting the PD-1/PD-L1 axis employed GL261, contributing to inflated expectations about the clinical efficacy of immune checkpoint blockade in GBM [26, 61, 62].

Concurrently, recent scientific and regulatory committees have increasingly emphasized reducing reliance on preclinical animal models, citing longstanding ethical concerns, limited translational relevance, and advancements *in vitro* and *ex vivo* model systems. The FDA has adopted initiatives that permit new approach methodologies, such as organoid modeling, to identify therapeutic candidates for clinical trials. Our approach aligns with this paradigm shift by bridging organoid and immunological research. By polarizing primary macrophages with patient-derived xenolines, our model enables incorporation of immune components into GBM organoid platforms, allowing for more physiologically accurate investigations of tumor-immune interactions. Indeed, our findings highlight the utility of xenoline-polarized macrophages as a more physiologically relevant TAM model relative to traditional IL-4/IL-13-polarized macrophages. Unlike IL-4/IL-13 polarized macrophages, xenoline-polarized TAMs exhibit a broader spectrum of transcriptional and functional states that more closely mirror the complexity of the tumor microenvironment. This includes simultaneous enrichment of inflammatory, hypoxic, and EMT signatures that are typically observed *in vivo*. Additionally, our co-culture models faithfully recreate some of the differences in macrophage gene signatures observed between primary and recurrent glioblastoma, notably our JX14P-RT TAMs exhibited an enrichment in the MAC_IFN subset. Conversely, JX14P TAMs displayed an enrichment of the MDM_Phagocytic subset, which was non-significantly enriched in primary patient samples. This further supports the relevance of our co-culture system in capturing the phenotypic diversity of TAMs across disease states. Importantly, because these polarization states are generated through tumor-secreted factors rather than artificial cytokine stimulation, they may more accurately reflect the immune programming that occurs within the glioma microenvironment. Taken together, our findings highlight xenoline-polarized macrophages as a versatile and translationally relevant model for investigating TAM function, therapeutic targeting, and immune adaptation during glioblastoma progression.

This model is not without its limitations. We suspect that our recurrent TAM model was not enriched for MAC_Metab_Hypoxia, MAC_Lipid_Metab, and to a lesser extent, MAC_Anti-Inflam subsets, partially due to the absence of a hypoxic niche in our model. Perhaps the addition of an ECM in a direct co-culture would allow for the formation of hypoxic niches in a tumoroid model. Also, it is important to note that the sequencing of our TMA includes transcripts from all cell types within the tumor, and thus observed signatures may reflect contributions from non-macrophages populations. Future studies must employ single cell sequencing methods to definitively quantify the relative proportion of each subset within the different polarization states to help elucidate what extracellular dependencies are necessary to better replicate TAM biology in the *in vitro* setting.

Additionally, we recognize that while GSEA provided useful predictions regarding macrophage function, several of our *in vitro* assays revealed discrepancies between the signatures and observed phenotypes. Although the MDM_Phagocytic gene set predicted the highest phagocytic capacity in the M1 macrophages and lowest in M2, the M2 macrophages exhibited the highest rate of phagocytosis against JX14P compared to JX14P TAMs and JX14P-RT TAMs. Additionally, M0 exhibited increased phagocytosis relative to JX14P-RT TAMs. Similarly, while JX14P-RT was enriched for EMT transition and promoted greater invasion than M0 and M2 macrophages, their invasion-promoting capacity did not significantly differ from JX14P TAMs and M1 macrophages.

Finally, despite multiple enrichments in MAC_Perivascular signatures in xenoline-polarized TAMs, HUVEC tube formation assay revealed no differences in angiogenic potential across polarization states. However, it must be noted that these discrepancies in the tube formation assay are not exclusively computationally related. The tube formation assay results may have been skewed for multiple reasons. First, macrophage capacity to support angiogenesis may be dependent upon additional parenchymal or stromal interactions, as previously reported [36]. Additionally, the proportion of CM used or the concentration of EGF and FGF in the basal media may have affected assay sensitivity. Altogether, the discrepancies in the functional assays illustrate a broader issue, the inconsistent discordance between transcriptomic predictions and functional phenotypes.

The discordance highlights the necessity for functional validation of gene sets and subtypes identified from sequencing data. A previous study stratified patients based on enrichment of six gene sets associated with radioresistance, hypoxia, G2M Checkpoint, Reactive Oxygen Species (ROS), UV Response Up, UV Response Down, and DNA Repair and performed a survival analysis across multiple cancer types, including cervical, head and neck, and breast cancers [13]. Interestingly, enrichment of a given gene set was variably associated with improved or worsened survival depending on the cancer type, highlighting the context dependent nature of these pathways and the need to validate the phenotype. In our patient-matched primary and recurrent GBM specimens, we observed enrichment of the Hypoxia and UV Response Down gene sets in recurrent GBM. Interestingly, our JX14P-RT TAM models were also significantly enriched for Hypoxia and UV Response Down gene sets, in the absence of radiation exposure, suggesting that this model captures key phenotypic differences between primary and recurrent GBM, and that tumor-intrinsic factors are sufficient to induce phenotypic changes in macrophages. These findings also raise the possibility that the xenoline JX14P-RT may prime macrophages to adopt a radiation-resistant phenotype. Future studies will investigate whether radiation-selected xenolines exert a radioprotective effect on TAMs, with the broader goal of better understanding how radiotherapy shapes the immune landscape of GBM.

As we continue to refine our understanding of TAM biology, establishing models that faithfully recapitulate the phenotypic and functional complexity of TAMs in the TME becomes increasingly important. This work underscores the limitations of IL-4/IL-13-stimulated macrophages and supports the adoption of xenoline-polarized macrophage models to improve the fidelity of preclinical GBM research. By more accurately modeling TAM biology, we can advance the development of innate immunomodulatory therapeutics in hopes of improving patient outcomes in GBM.

## Data Availability

The proteomic dataset supporting the conclusions of this article is available in the EMBL-EBI ArrayExpress Archive repository, (accession # and hyperlink to dataset pending). The Sequencing datasets supporting the conclusions of this article are available in the Gene Expression Omnibus repository, (accession #s and hyperlinks to datasets pending). Code is available upon request.
